
JOURNAL INFORMATION
==============================
NLM Title Abbreviation: Wirtschaftsdienst
Journal ID: Wirtschaftsdienst
NLM Journal ID: 101086612

Wirtschaftsdienst (Hamburg, Germany : 1949)

ISSN: 0043-6275

ARTICLE INFORMATION
==============================
PMCID: PMC7088298
PMID: 32214514
DOI: 10.1007/s10273-020-2607-5
Article ID: 2607
Article version: 1
Subjects: Ökonomische Trends

Coronavirus hält Weltkonjunktur in Atem

Business cycle highlight: Coronavirus keeps the global economy in suspense

Gern Klaus-Jürgen klaus-juergen.gern@ifw-kiel.de
1
Hauber Philipp philipp.hauber@ifw-kiel.de
2
1 grid.462465.7 0000 0004 0493 2817 Forschungsgruppe Intern. Konjunktur, Institut für Weltwirtschaft, Kiellinie 66, 24105 Kiel, Deutschland
2 grid.462465.7 0000 0004 0493 2817 Prognosezentrum, Institut für Weltwirtschaft, Kiellinie 66, 24105 Kiel, Deutschland
Electronic publication date: 2020 Mar 23
Print publication date: 2020
Volume: 100
Issue: 3
First page: 223
Last page: 224
Copyright: © Der/die Autor(en) 2020
License: Open Access Dieser Artikel wird unter der Creative Commons Namensnennung 4.0 International Lizenz veröffentlicht, welche die Nutzung, Vervielfältigung, Bearbeitung, Verbreitung und Wiedergabe in jeglichem Medium und Format erlaubt, sofern Sie den/die ursprünglichen Autor(en) und die Quelle ordnungsgemäß nennen, einen Link zur Creative Commons Lizenz beifügen und angeben, ob Änderungen vorgenommen wurden.
Die in diesem Artikel enthaltenen Bilder und sonstiges Drittmaterial unterliegen ebenfalls der genannten Creative Commons Lizenz, sofern sich aus der Abbildungslegende nichts anderes ergibt. Sofern das betreffende Material nicht unter der genannten Creative Commons Lizenz steht und die betreffende Handlung nicht nach gesetzlichen Vorschriften erlaubt ist, ist für die oben aufgeführten Weiterverwendungen des Materials die Einwilligung des jeweiligen Rechteinhabers einzuholen.
Weitere Details zur Lizenz entnehmen Sie bitte der Lizenzinformation auf http://creativecommons.org/licenses/by/4.0/deed.de.
License URL: https://creativecommons.org/licenses/by/4.0/

issue-copyright-statement: © The Author(s) 2020

==============================
DOI: 10.1007/s10273-020-2607-5

Konjunkturschlaglicht

### Coronavirus hält Weltkonjunktur in Atem

Die auf den Erreger SARS-CoV-2 zurückzuführende Lungenkrankheit Covid-19 trat erstmals Ende 2019 in der chinesischen Stadt Wuhan auf und breitete sich in der Folge zunächst in China, vor allem in der Provinz Hubei, aus. Seit Februar werden zunehmend Infektionen außerhalb Chinas verzeichnet (vgl. Abbildung 1). Am 10. März 2020 berichtete die Weltgesundheitsorganisation (WHO) knapp 120 000 bestätigte Fälle in mehr als 100 Ländern, mit knapp 4000 Toten und Schwerpunkten außerhalb Chinas in Südkorea, Iran und Italien.

Studien zu den wirtschaftlichen Auswirkungen von Epidemien rekurrieren zumeist auf die Effekte, die direkt aus einer Reduzierung des Arbeitsangebots durch höheren Krankenstand und durch krankheitsbedingte Todesfälle resultieren. Für die Konjunktur sind aber indirekte Wirkungen, die von Maßnahmen zur Eindämmung der Epidemie oder Verhaltensänderungen der Konsumenten ausgehen, bislang von größerer Bedeutung. Diese beeinträchtigen die wirtschaftliche Aktivität unmittelbar dort, wo viele Menschen auf engem Raum zusammentreffen, etwa durch die Absage von Großveranstaltungen und Messen, und führen zu einem Rückgang im Reiseverkehr. Quarantänemaßnahmen können darüber hinaus die Warenproduktion behindern und die Produktionsabläufe – auch grenzüberschreitend – empfindlich stören.

Bereits früh zeichnete sich ab, dass die Zahl der Infizierten deutlich höher liegen würde als bei dem SARS-Ausbruch im Jahr 2003.[1] Infolge der SARS-Epidemie kam es im ersten Quartal 2003 zu einer moderaten Abnahme der gesamtwirtschaftlichen Produktion in China und zu einem spürbaren Rückgang im innerasiatischen Flugverkehr; insgesamt blieben die Auswirkungen allerdings überschaubar. Dass die wirtschaftlichen Konsequenzen der Epidemie dieses Mal erheblich größer sind, liegt allerdings weniger an der größeren Zahl der Infektionen und Todesfälle. Wichtiger ist, dass die Behörden in China wesentlich drastischer reagierten, um die Ausbreitung des Virus einzudämmen.[2] In der übrigen Welt sind die Auswirkungen zum einen deshalb größer, weil China als Bestandteil der internationalen Wertschöpfungsketten und als Absatzmarkt stark an Bedeutung gewonnen hat – so erhöhte sich der Anteil Chinas an der Weltproduktion von gut 4 % im Jahr 2003 auf mehr als 16 % im Jahr 2019, der Anteil Chinas am Welthandel von 5 % auf 12,4 %.

Abbildung 1
**Covid-19-Fälle in China und übrigen Ländern**
Tagesdaten. Letzter Wert: 10.3.2020

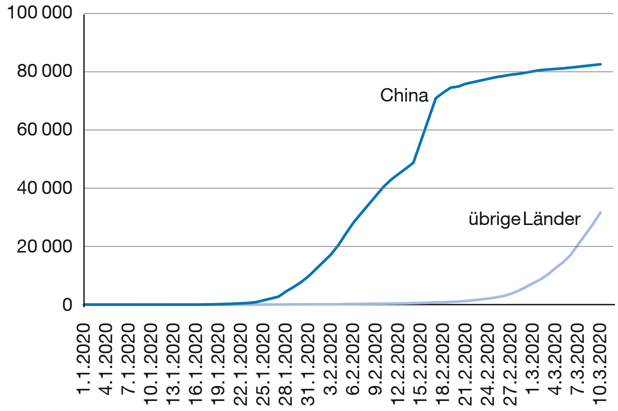

Quelle: Weltgesundheitsorganisation.

### Temporäre Schockstarre in China

In der am stärksten betroffenen Provinz Hubei, die etwa 4 % der chinesischen Wirtschaftsleistung ausmacht und einen leicht überdurchschnittlichen Wertschöpfungsanteil des Produzierenden Gewerbes aufweist, kam es Ende Januar 2020 zu weitreichenden Quarantänemaßnahmen. Die Verkehrsverbindungen zum Rest des Landes wurden gekappt, Schulen und Produktionsstätten geschlossen und die Bewegungsfreiheit erheblich eingeschränkt. Aber auch im Rest des Landes kam es zu Reisebeschränkungen; landesweit wurden alle Kinos geschlossen und Feierlichkeiten zum Neujahrsfest abgesagt. Zudem wurden die Neujahrsferien verlängert.

Die Auswirkungen auf die gesamtwirtschaftliche Aktivität waren drastisch. Zeitnah verfügbare Indikatoren wie Kohleverbrauch, Transportvolumen oder das mithilfe von Staudaten geschätzte Verkehrsaufkommen legen nahe,

© Der/die Autor(en) 2020. Open Access: Dieser Artikel wird unter der Creative Commons Namensnennung 4.0 International Lizenz (https://creativecommons.org/licenses/by/4.0/deed.de) veröffentlicht.

Open Access wird durch die ZBW – Leibniz-Informationszentrum Wirtschaft gefördert.

1 Im Zuge der SARS-Epidemie zwischen Herbst 2002 und Frühjahr 2003 infizierten sich laut WHO weltweit etwas mehr als 8000 Menschen, bei einer allerdings deutlich höhen Sterblichkeitsrate von etwa 10 %.
2 Vgl. R. Döhrn: Auswirkungen der COVID-19 Epidemie auf die chinesische Wirtschaft – eine erste Abschätzung, RWI Materialien, Nr. 134, 2020.

Abbildung 2
**Einkaufsmanagerindizes in China**
Indizes (monatlich)

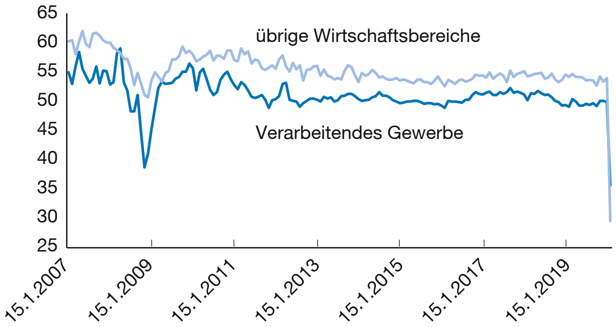

Quelle: Nationales Statistikbüro der Volksrepublik Chinas.

dass die Produktion massiv beeinträchtigt wurde. Nachfrageseitig lässt der für Februar 2020 gemeldete Rückgang der Autoverkäufe um mehr als 90 % gegenüber dem Vorjahr darauf schließen, wie stark die wirtschaftliche Aktivität beeinträchtigt war. Für Februar liegen inzwischen auch die Befragungen von Einkaufsmanagern vor. Der auf dieser Basis ermittelte Stimmungsindex brach dramatisch ein und fiel im Verarbeitenden Gewerbe auf ein ähnliches Niveau wie während der großen Rezession 2008, in den übrigen Wirtschaftsbereichen sogar darunter (vgl. Abbildung 2). Zwar wurden in der zweiten Februarhälfte vielerorts die Restriktionen gelockert und in vielen Betrieben die Produktion wieder aufgenommen. Von einer normalen Auslastung ist die chinesische Wirtschaft allerdings noch weit entfernt. Immerhin sind die meisten Wanderarbeiter wieder an ihre Arbeitsplätze zurückgekehrt, sodass die Produktion von dieser Seite wieder möglich wird.

Für das erste Quartal liegt der durch das Coronavirus verursachte Rückgang von Investitionen und Konsum in China nach derzeitigen Prognosen in einer Größenordnung von 4 % – dies führt zu einem Rückgang der Zuwachsrate des BIP gegenüber dem Vorjahr von 6 % auf 3,5 %. Unter der Annahme, dass sich die Produktion im Laufe des zweiten Quartals allmählich wieder dem Normalniveau annähert, ergibt sich eine Verminderung der Zuwachsrate des chinesischen BIP im Jahr 2020 insgesamt um 1,3 %.[3] Die jüngsten Indikatoren lassen aber auch eine deutlich stärkere Schrumpfung der Wirtschaft möglich erscheinen.

### Starker Dämpfer für die Weltkonjunktur

Die Produktion außerhalb Chinas wird zum einen durch die drastischen Rückgänge von Produktion und Nachfrage in China selbst gedämpft. Die durch die Verflechtungen im Handel und Kapitalverkehr bedingte internationale Übertragung von Schocks auf Produktion und Nachfrage können mithilfe von Simulationsrechnungen in makroökonomischen Weltmodellen abgebildet werden. Demnach führt allein die verringerte Produktion in China zu einem Rückgang der Produktion in der übrigen Welt um 0,3 %, mit besonders ausgeprägten Effekten im asiatischen Raum. In Deutschland würde die Produktion um knapp 0,2 Prozentpunkte reduziert. Dabei sind die gravierenden Einbußen im Tourismus vermutlich nur unzureichend abgebildet. Dies gilt vor allem in Asien, wo Reisende aus China einen Anteil von bis zu einem Drittel ausmachen. Auch die Unterbrechung von Lieferketten wird die Probleme für die Produktion außerhalb Chinas noch verstärken.

Vor allem aber werden im Zuge der globalen Ausbreitung der Epidemie auch außerhalb Chinas zunehmend vorbeugende Maßnahmen ergriffen, welche die wirtschaftliche Aktivität bremsen. Durch das veränderte Verhalten der Konsumenten dürften (auch in anderen Ländern) insbesondere der Dienstleistungssektor eingebremst werden; nicht zuletzt in den Ländern, die stark vom Tourismus abhängen. Zudem dürfte die Unsicherheit über Ausmaß und Dauer der Krise zu einer zeitweisen Investitionszurückhaltung führen.

Gemäß Modellsimulationen verändert sich die Zuwachsrate der Weltproduktion nur unwesentlich, wenn sich die verminderte wirtschaftliche Aktivität durch Maßnahmen zur Seuchenbekämpfung auf wenige andere Länder wie Korea oder Italien beschränkt. Erheblich sind zusätzliche Belastungen aber dann, wenn solche Maßnahmen verbreitet angewendet werden müssen oder der Einbruch der Produktion in China wesentlich stärker ausfällt, als in dem oben skizzierten Szenario unterstellt.

Inzwischen werden in vielen Ländern Maßnahmen ergriffen, um die Wirtschaft zu stützen. Gezielte finanzpolitische Programme, Kreditgarantien und einkommensstabilisierende Zahlungen (wie in Deutschland das Kurzarbeitergeld) sind zwar nicht in der Lage, die kurzfristigen Auswirkungen der Epidemie auf Produktion und Nachfrage nennenswert zu verringern, sie können aber Liquiditätsproblemen begegnen, einer zyklischen Verstärkung der Nachfrageschwäche entgegenwirken und längerfristige negative Folgen verhindern, die durch Unternehmenszusammenbrüche und Arbeitsplatzverluste für das Produktionspotenzial entstehen könnten.

Klaus-Jürgen Gern, Philipp Hauber
Klaus-Juergen.Gern@ifw-kiel.de, philipp.hauber@ifw-kiel.de

3 Für eine solche Simulationsrechnung vgl. OECD Interim Economic Assessment, 2. März 2020.
